# Angiopoietin-like Protein 3 (ANGPTL3) Targeting in the Management of Dyslipidemias

**DOI:** 10.3390/ijms27020921

**Published:** 2026-01-16

**Authors:** Constantine E. Kosmas, Loukianos S. Rallidis, Ioannis Hoursalas, Evangelia J. Papakonstantinou, Christina E. Kostara

**Affiliations:** 1 2nd Department of Cardiology, National & Kapodistrian University of Athens, 12462 Athens, Greece; cekosmas1@gmail.com (C.E.K.); lrallidis@gmail.com (L.S.R.); 2 School of Medicine, European University Cyprus, 2404 Nicosia, Cyprus; ihoursal@otenet.gr; 3 General Directorate of Public Health and Social Welfare, Attica Region, 11521 Athens, Greece; lpapakon777@gmail.com; 4 Laboratory of Clinical Chemistry, Faculty of Medicine, School of Health Sciences, University of Ioannina, 45110 Ioannina, Greece

**Keywords:** dyslipidemia, angiopoietin-like 3, ANGPTL3 inhibitors, evinacumab, antisense oligonucleotides (ASOs), gene editing, small interfering RNAs (siRNAs), zodasiran, solbinsiran, CRISPR-Cas systems

## Abstract

Cardiovascular disease (CVD) remains the leading cause of morbidity and mortality, despite advances in pharmacological prevention and treatment. The burden of CVD necessitates implementing the treatment of risk factors including dyslipidemia. Pharmaceutical advancements and in depth understanding of pathophysiology have enabled innovative therapies targeting pathways underlying lipoprotein metabolism disorders. Angiopoietin protein-like 3 (ANGPTL3) plays a crucial role in the regulation of lipoprotein metabolism, therefore being a potential therapeutic target. Inhibition of ANGPTL3 has emerged as a new therapeutic strategy to reduce LDL-cholesterol levels independent of the LDL receptor function. Therapeutic approaches for ANGPTL3 inhibition range from monoclonal antibodies to nucleic acid therapeutics including antisense oligonucleotides and small interfering RNAs. In this review, we briefly explain the structure and mechanism of action of ANGPTL3 and discuss the therapeutic approaches for targeting ANGPTL3 in the clinical setting. We also discuss Evinacumab, a monoclonal antibody, its structure, mechanism of action, safety, tolerability, pharmacokinetics, and pharmacodynamics, as well as its clinical trial-derived results. The antisense oligonucleotides modify ANGPTL3 mRNA to inhibit protein production, and small interfering RNAs induce mRNA degradation; results from clinical trials were reviewed in detail. Finally, we discuss promising gene editing approaches including clustered regularly interspaced short palindromic repeats (CRISPR)/Cas systems.

## 1. Introduction

Cardiovascular disease (CVD) stands as the foremost leading cause of morbidity and mortality globally, representing a major contributor to the global burden of disease and healthcare expenditures, despite substantial advances in pharmacological strategies for its prevention and management [1,2]. Between 1990 and 2021, the incidence of CVD cases increased from 34.74 million to 66.81 million, while CVD-related deaths increased from 12.33 million to 19.42 million during the same period, reflecting a 92.3% increase in cases and a 57.5% rise in mortality [3]. To mitigate the increasing global burden of CVD [4], public health organizations have focused on reducing major modifiable CVD risk factors, including hypertension, obesity, and dyslipidemia.

Dyslipidemia is characterized by abnormal lipid and lipoprotein profiles in the bloodstream due to impaired regulation of lipid metabolism. Higher atherogenic lipid burden (including elevated concentrations of total cholesterol, LDL-cholesterol, and non-HDL-cholesterol, as well as increased serum triglycerides (TG) and/or diminished HDL-cholesterol levels) is associated with a graded increase in CVD events. Abnormal serum lipid levels are strongly associated with CVD; especially, elevated levels of LDL-cholesterol is a well-known established causal factor of atherosclerotic cardiovascular disease (ASCVD) [5]. In 2021, 3.81 million cardiovascular deaths and 3.81 million deaths overall were attributed to elevated LDL-cholesterol levels [4].

The prevalence of dyslipidemia has increased in the past 30 years [6]. According to World Health Organization (WHO), over 4 million deaths per year are associated with dyslipidemia. Also, according to the American Heart Association’s 2024 Heart Disease and Stroke Statistics Report (for the period 2017 to 2020), the prevalence rates in male and female adults, respectively, did not differ significantly for the elevated total cholesterol (total cholesterol ≥ 200 mg/dL: 32.8% in males, 36.2% in females) and LDL-cholesterol (LDL-cholesterol ≥ 130 mg/dL: 25.6% in males, 25.4% in females), whereas decreased HDL-cholesterol concentrations (<40 mg/dL) were more common in males (25%) than females (9.3%). Increased TG concentrations occurred in 19.9% of adults. For 2021, data indicate an age-adjusted mortality rate of 237.9 per 100,000 individuals due to CVD, with elevated LDL-cholesterol concentrations contributing to an estimated 3.72 million deaths [7]. There is thus a clear need to lower plasma lipid levels to battle the worldwide cardiovascular disease burden. Dyslipidemia represents a compelling area for the exploration of novel management strategies [8].

Statins, or 3-hydroxy-3-methylglutaryl coenzyme A (HMG-CoA) reductase inhibitors, are widely established as the first-line therapy for people displaying elevated plasma LDL-cholesterol levels. These agents exert their primary lipid-lowering effect by significantly reducing LDL-cholesterol levels. Although LDL-cholesterol is a well-established causal factor for the development of ASCVD, it does not reflect the total plasma atherogenicity accounting for all apolipoprotein B (apoB)-containing lipoproteins, which also include very low-density lipoprotein (VLDL), intermediate density lipoprotein (IDL), their remnants, and lipoprotein (a) [Lp(a)]. Recent studies have provided insights into the genetic origin of dyslipidemias, as well as their role in the development of atherosclerosis [7]. Progress in genetics and analytical tools of signaling molecules including PCSK9, ANGPTL3, ANGPTL4, APOC3, and LPA has revealed a wide range of new mechanisms that can be targeted for therapy to reduce cholesterol levels. Moreover, progress has been made in developing advanced methods that use small molecules, biological agents, or nucleic acids to target these recently identified mediators. New treatment strategies targeting hepatocytes that are involved in lipid metabolism, have been made possible by these technologies [9].

As dyslipidemia continues to be a critical risk factor for CVD, this review seeks to highlight the promise of a more targeted, personalized, efficient, and safer alternative in reducing the global burden of cardiovascular diseases. We discuss the structure and mechanism of action of angiopoietin-like protein 3 (ANGPTL3) and the therapeutic approaches for targeting ANGPTL3 in the clinical setting. We also discuss the current therapeutic options including monoclonal antibodies, antisense oligonucleotides modify mRNA and small interfering RNAs, as well as their clinical efficacy in dyslipidemia.

## 2. Angiopoietin-like Protein 3 (ANGPTL3): Structure and Function

The angiopoietin-like proteins (ANGPTLs) compose a specific family of secreted glycoproteins sharing a structural similarity to angiopoietins, which have a critical role in the physiology of angiogenesis [10]. To date, eight ANGPTLs (ANGPTL1-8) have been identified, which are composed of an amino (N)-terminal coiled-coil domain, a linker region, and a carboxy (C)-terminal fibrinogen-like domain. Although they share common structural characteristics, the eight ANGPTLs present distinct physiological functions in angiogenesis, inflammation, tissue remodeling, and lipid metabolism [11]. Especially, ANGPTL3, ANGPTL4, and ANGPTL8 are characterized by high sequence homology with a pivotal role in lipoprotein homeostasis and lipid-induced related diseases. Studies have shown that they affect plasma lipid levels by inhibiting extracellular lipases, including lipoprotein lipase (LPL), hepatic lipase, endothelial lipase (EL), and pancreatic lipase, leading to increased circulating levels of TG, LDL-cholesterol, and HDL-cholesterol [12,13].

**Figure 1:**
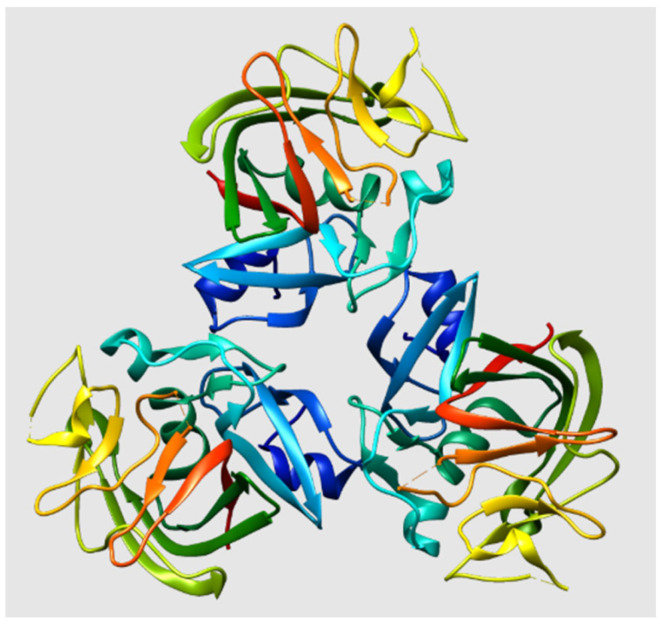
Structure of ANGPTL3 protein. From “Visualisation de la protéine Cristallisée ANGPTL3 à partir de l’identifiant PDB 6EUA sur le logiciel UCSF Chimera” [17] CC BY.

ANGPTL3 is a member of ANGPTL protein family, which was identified independently around the year 2000. ANGPTL3, encoded by the *ANGPTL3* gene on chromosome 1, is a 70 kDa secreted glycoprotein consisting of 460 amino acids and is mainly expressed in the liver during both embryonic and adult stages (Figure 1). A coiled-coil structure between N-terminal coiled-coil domain and C-terminal globular fibrinogen homology domain [14] is required for the protein’s main function, namely the cleavage and inhibition of LPL and EL activity. Specifically, an N-terminal signal peptide is responsible for secretion, an N-terminal alpha helix (the amino acid domain 61–66) affects plasma TG levels via reversibly inhibiting catalytic activity of triglyceride lipases including LPL and EL [15], a C-terminal fibrinogen-like domain (FLD) (207–460 amino acid domain) is involved in angiogenesis [16], and a short linker region (at 221–222 and 224–225 amino acid domain) between N- and C-terminal domains functions as a site of cleavage that is required for protein activation.

The function of ANGPTL3 in the regulation of lipid metabolism is associated with the inhibition of LPL and EL, two enzymes playing an important role in lipoprotein metabolism. LPL is an enzyme that plays a critical role in hydrolyzing TG carried by VLDL and chylomicron (CM) particles in the bloodstream; whereas, EL hydrolyzes HDL-phospholipids and consequently decreases plasma HDL-cholesterol levels through a putative heparin-binding site in the N-terminal domain of ANGPTL3. The association of ANGPTL3 with lipid metabolism was discovered in 2002 via identification of an ANGPTL3-mutant KK/San hypertriglyceridemic and hyperglycemic obese mouse strain characterized by abnormally low TG levels [18] and elevated post-heparin LPL activity [12]. Since then, many animal studies have confirmed that ANGPTL3 serves as an inhibitor of LPL and thereby reduces plasma triglyceride clearance, partially explaining the hypolipidemic phenotype of the KK/San mice [12,18]. Along with its ability to inhibit LPL enzymatic activity, ANGPTL3 also suppresses a second intravascular lipase, EL, promoting the clearance of high levels of VLDL remnants via the VLDL receptor and other receptors, including LDL receptor-related proteins. This mechanism explains the LDL-cholesterol-lowering effect of ANGPTL3 inhibition and is independent of the LDL receptor.

Consistent with these findings, genome-wide association studies (GWAS), as well as dedicated studies of common variants, have identified single-nucleotide polymorphisms in and near the ANGPTL3 gene locus with significant effects on lipid metabolism (association of ANGPTL3 with plasma triglyceride and plasma HDL cholesterol levels), thus confirming the vital role of ANGPTL3 in lipid metabolism and laying the foundations for the development of ANGPTL3 inhibitors [19,20,21,22,23,24,25].

## 3. Targeting ANGPTL3 in the Clinical Setting

ANGPTL3-targeted therapies offer a novel, LDL receptor-independent mechanism to manage lipid disturbances, particularly for patients with severe or genetically driven dyslipidemias. While monoclonal antibodies including evinacumab, SHR1918 [26], and RNA-based (ASOs and siRNAs) agents including zodasiran and solbinsiran are closer to everyday clinical use, gene-editing approaches such as CTX310 may redefine long-term treatment paradigms pending confirmation of safety and durability (Table 1).

### 3.1. Monoclonal Antibody Targeting ANGPTL3, Evinacumab

ANGPTL3 inhibitors agents constitute novel medications, which inhibit the activity of ANGPTL3 or its production from hepatocytes in order to imitate the phenotype of loss-of-function mutations. Evinacumab is a monoclonal antibody that binds to, and pharmacologically inhibits ANGPTL3, which was approved by the US Food and Drug Administration (FDA) on February 2021 and the European Medicines Agency (EMA) in June 2021, and is now available to treat adult and adolescent patients (≥12 years) with homozygous familial hypercholesterolemia. The recommended dose of this drug is 15 mg/kg, administered by intravenous infusion (IV) over one hour once monthly.

***Structure:*** From the structure point of view, evinacumab is an IgG4-kappa monoclonal antibody with a Y-shaped morphology. It consists of two identical heavy chains (453 amino acids each in length) and two identical kappa light chains, each comprising 214 amino acids. The heavy chains are covalently linked with disulfide bonds at their hinge region [27].

***Mechanism of action:*** After subcutaneous (SC) or intravenous (IV) administration, evinacumab binds to its target, ANGPTL3, which, as described above, prevents the LPL- and EL-mediated lipid hydrolysis. As a result, the inhibition of ANGPTL3 by evinacumab inhibits its function leading to increased activities of LPL and EL enzymes and lower levels of TG, LDL-cholesterol, and HDL-cholesterol independently of LDL receptor function.

***Safety, tolerability, pharmacokinetics, and pharmacodynamics:*** A randomized, double-blind, placebo-controlled, Phase 1 clinical trial was conducted in order to assess the safety, tolerability and pharmacokinetics of evinacumab [28]. Ninety-six healthy Caucasian and Japanese volunteers with LDL-cholesterol levels between 100 and 160 mg/dL were enrolled in the study. Participants were randomly assigned in four different cohorts: evinacumab SC 300 mg (single dose), SC 300 mg (once weekly for eight doses), IV 5 mg/kg, or IV 15 mg/kg (once every 4 weeks for two doses). Each cohort comprised 24 participants, 12 Japanese and 12 Caucasian, to receive evinacumab or placebo with a 24-week follow-up. Evinacumab was found to be well tolerated at all dose regimens, including the highest administered doses of 15 mg/kg IV every 4 weeks and 300 mg SC in a weekly basis. The safety profile of evinacumab was comparable to that of placebo in both ethnicities, with no serious treatment-emergent adverse events or adverse events leading to study discontinuation. In addition, the pharmacokinetic and pharmacodynamic profiles of evinacumab were similar in both ethnicities at all dose regimens. From a pharmacodynamic perspective, evinacumab administration resulted in significant, dose-dependent reductions in LDL-cholesterol, TG, and apolipoprotein B (apoB) levels in all subjects [28].

***Clinical Trials:*** Several clinical trials have evaluated the efficacy of the monoclonal antibody evinacumab in lowering LDL cholesterol levels in patients with severe and refractory hypercholesterolemia (Table 2).

Gaudet et al. conducted a single-group, open-label, proof-of-concept, Phase 2 clinical trial (NCT02265952) in order to evaluate the efficacy and safety of evinacumab in patients with homozygous familial hypercholesterolemia (HoFH) [29]. The studied group included nine HoFH participants receiving maximum-tolerated lipid-lowering treatment. Participants received evinacumab treatment for a total of four weeks. After the 4 week evinacumab treatment, there was a mean reduction in LDL-cholesterol by 49 ± 23%, with an absolute decrease from baseline of 157 ± 90 mg/dL. In addition, apoB, non-HDL-cholesterol, TG, and HDL-cholesterol were reduced by 46 ± 18%, 49 ± 22%, 47%, and 36 ± 16%, respectively. According to the authors, all nine patients reported the occurrence of at least one adverse event, but no event led to treatment discontinuation [29].

Banerjee et al. [30] analyzed the effects of evinacumab on LDL receptor activity in lymphocytes purified from patients in the aforementioned Phase 2, proof-of-concept study (NCT02265952), which demonstrated that evinacumab reduced LDL-cholesterol levels in nine patients with genotypically confirmed homozygous familial hypercholesterolemia and was well tolerated. Based on the results, the authors suggested that evinacumab is effective for lowering LDL-cholesterol in patients with homozygous familial hypercholesterolemia, and the inhibition of ANGPTL3 in humans lowers LDL-cholesterol in a mechanism independent of the LDL receptor [30].

Rosenson et al. [31] carried out a double-blind, placebo-controlled, Phase 2 trial across 20 countries (NCT03175367). This study enrolled 272 patients with hypercholesterolemia refractory to treatment with a PCSK9 inhibitor and a maximally tolerated statin dose, with or without ezetimibe. The screening LDL-cholesterol concentration was 70 mg/dL or higher for patients with clinical ASCVD and 100 mg/dL or higher for patients without clinical ASCVD. Patients were randomly assigned to receive SC or IV evinacumab or placebo for a period of 16 weeks. At week 16, the differences from baseline in the LDL-cholesterol between the groups receiving SC evinacumab and placebo ranged from −38.5% to −56.0% depending on the received dose regimen. The respective differences between the groups receiving IV evinacumab and placebo ranged from −24.2% to −50.5% depending on the administered dose. A similar incidence of adverse events was observed in both the SC and IV evinacumab groups, with serious adverse events during the treatment period ranging from 3% to 16% across the trial cohorts. Evinacumab significantly reduced LDL-cholesterol by more than 50% at the maximum dose [31].

Reeskamp LF et al. [32] (NCT04722068) investigated the apoB containing lipoproteins kinetics before and after evinacumab administration in four patients with HoFH already participating in the above-mentioned study (NCT02265952). A stable isotope (5,5,5-2H3)-Leucine was administrated and measured in VLDL, IDL, and LDL before and after the IV administration of evinacumab 15 mg/kg. Evinacumab treatment lowered levels of LDL-cholesterol by 59 ± 2% and increased the fractional catabolic rate of IDL-apoB and LDL-apoB in all 4 HoFH subjects, by 616 ± 504% and 113 ± 14%, respectively. VLDL-apoB production rate decreased in two of the four subjects. The authors concluded that the evinacumab-mediated inhibition of ANGPTL3 was associated with an increase in the fractional catabolic rate of IDL-apoB and LDL-apoB, suggesting that evinacumab lowers LDL-cholesterol levels primarily by increasing the clearance of apoB-containing lipoprotein from the circulation [32].

Additionally, Reeskamp et al. [33] conducted a double-blind, placebo-controlled, Phase 3 trial in order to investigate whether intensive lipid-lowering therapy with evinacumab in two adolescents with null/null variants, aged 12 (patient A) and 16 years (patient B), might result in atherosclerotic plaque regression. Coronary computed tomography angiographies (CCTAs), before randomization and after a period of 6 months of treatment with evinacumab, showed that total plaque volumes were lowered by 76% and 85% in patients A and B, respectively. The authors concluded that the formation of atherosclerotic plaque may be a potentially reversible process [33].

In a meta-analysis of five randomized controlled trials (RCTs) with a total of 568 subjects, treatment with evinacumab significantly reduced LDL-cholesterol [mean difference (MD) −33.123%, 95% confidence interval (CI), −48.639% to −17.606%, *p* < 0.0001], triglycerides (MD −50.959%, 95% CI, −56.555% to −45.362%, *p* < 0.0001), and HDL-cholesterol (MD −12.773%, 95% CI, −16.359% to −9.186%, *p* < 0.0001) compared with placebo [34]. According to the authors, the incidence of at least one treatment-emergent adverse event was not significantly different between evinacumab and placebo groups (relative risk 1.080, 95% CI, 0.901–1.296, *p* = 0.405). The authors concluded that evinacumab decreased TG, LDL-cholesterol, and HDL-cholesterol without significant adverse effects, indicating that it can be a therapeutic choice for lowering LDL-cholesterol [34].

Raal FJ et al. evaluated longer-term efficacy and safety of evinacumab in 64 patients with HoFH from the ELIPSE HoFH study and received intravenous evinacumab 15 mg/kg every 4 weeks [35]. The mean baseline LDL-cholesterol was 250.5 ± 162.3 mg/dL at baseline; whereas, by week 48, evinacumab reduced mean LDL-cholesterol by 46.3%, with similar mean LDL-cholesterol reductions for patients with null-null (47.2%) and non-null variants (45.9%). Adverse events occurred in 47 (73.4%) patients; 4 (6.3%) patients experienced adverse events considered evinacumab-related (drug hypersensitivity, infusion-related reaction and asthenia, generalized pruritis, and muscle spasms). The authors concluded that in patients with HoFH, evinacumab demonstrated substantial and sustained LDL-cholesterol reduction regardless of LDL receptor function and was generally well tolerated.

Wiegman A et al. [36] conducted a three-part, single-arm, open-label clinical trial (NCT04233918) in order to evaluate the efficacy and safety of evinacumab in pediatric patients with HoFH with LDL-cholesterol > 130 mg/dL, despite optimized lipid-lowering therapy (including LDL receptor-independent apheresis and lomitapide). According to the authors, evinacumab treatment rapidly and durably (through week 24) decreased LDL-cholesterol with profound reduction in the first week, with a mean LDL-cholesterol reduction of −48.3% (10.4%) from baseline to week 24. Moreover, apoB (mean −41.3% [9.0%]), non-high-density lipoprotein cholesterol (−48.9% [9.8%]), and total cholesterol (−49.1% [8.1%]) were similarly significantly decreased [7]. Treatment-emergent adverse events were reported in 10 (71.4%) patients; however, only 2 (14.3%) reported events were considered to be treatment-related (nausea and abdominal pain). One serious treatment-emergent adverse event of tonsillitis occurred (n = 1), but this was not considered treatment-related. The authors concluded that evinacumab constitutes a new treatment for pediatric patients with HoFH and inadequately controlled LDL-cholesterol despite optimized lipid-lowering therapy, lowering LDL-cholesterol levels by nearly half in these extremely high-risk and difficult-to-treat individuals [36].

**Table 2:** Summary of the results of the clinical studies pertaining to evinacumab.

	Clinical Trial	Study Design	Results
Gaudet D et al. *The New England Journal of Medicine* **2017** [29]	Phase 2 NCT02265952	Single-group, open-label, proof-of-concept, Phase 2 clinical trial 9 HoFH participants 4 week evinacumab treatment	reduction in LDL-cholesterol 49 ± 23% apoB 46 ± 18% non-HDL-C 49 ± 22%, TG 47% HDL-cholesterol 36 ± 16%
Rosenson RS et al. *The New England Journal of Medicine* **2020** [31]	Phase 2 NCT03175367	Double-blind, placebo-controlled, Phase 2 trial across 20 countries 272 patients with hypercholesterolemia Treatment sc or iv evinacumab or placebo for 16 weeks.	LDL-C reduction: 56.0%: with 450 mg/w sc 52.9%: with 300 mg/w sc 38.5%: with 200 mg/2w sc 50.5%: with 15 mg/kg/Q4W iv 24.2%: with 5 mg/kg/Q4W iv
Reeskamp LF et al. *Arteriosclerosis, thrombosis, and Vascular Biology* **2021** [32]	Phase 2 NCT04722068	Lipoprotein kinetics of subjects enrolled in the R1500-CL-1331 clinical trial (NCT02265952) to assess the mechanism by which the evinacumab may affect lipid levels in HoFH subjects	LDL-cholesterol reduction: 59 ± 2% Increased IDL apoB and LDL apoB fractional catabolic rate: 616 ± 504% and 113 ± 14%
Wiegman A et al. *Circulation* **2024** [36]	Phase 3 NCT04233918	Pediatric patients with HoFH with LDL-C > 130 mg/dL	Reduction in LDL-cholesterol −48.3% from baseline to week 24. Reduction in apoB −41.3%, non-high-density lipoprotein cholesterol −48.9%, and total cholesterol −49.1%
Gaudet D et al. *European Heart Journal* **2024** [37]	Phase 3 NCT03409744	Open-label, single-group, Phase 3 clinical trial at 38 sites across 12 countries 116 HoFH patients 15 mg/kg intravenous every 4 weeks	Reduction in LDL-cholesterol: 43.6% in the overall population 41.7% and 55.4% in adults and adolescents. Evinacumab well tolerated.

Gaudet D et al. [37] conducted an open-label, single-group, Phase 3 clinical trial at 38 sites across 12 countries (NCT03409744) to assess the long-term safety and efficacy of evinacumab in 116 patients aged ≥12 years (adults: n = 102, adolescents: n = 14) with HoFH, who were evinacumab-naïve or had previously received evinacumab in other trials (evinacumab-continue). All received intravenous evinacumab 15 mg/kg every 4 weeks with stable lipid-lowering therapy. The authors reported that from baseline to Week 24, evinacumab decreased mean LDL-cholesterol by 43.6% in the overall population and mean LDL-C reduction in adults and adolescents was 41.7% and 55.4%, respectively. Treatment-emergent adverse events (TEAEs) and serious TEAEs were reported in 93 (80.2%) and 27 (23.3%) patients, respectively. Two (1.7%) deaths were reported (neither was considered related to evinacumab). Three (2.6%) patients discontinued due to TEAEs (none were considered related to evinacumab). The authors concluded that evinacumab was generally well tolerated and markedly decreased LDL-cholesterol irrespective of age and sex [37].

Evinacumab was approved in the United States since 11 February 2021, as a complementary agent to other LDL-cholesterol lowering therapies for patients aged 12 or older with HoFH [38]. It is worth noting that evinacumab is already approved for familial hypercholesterolemia by the FDA and EMA, whereas the other ANGPTL3 agents are still in the investigational stage, and, although initial trials appear promising, these agents have not yet been approved by the FDA or EMA.

### 3.2. RNA-Based Agents Targeting ANGPTL3

#### 3.2.1. Antisense Oligonucleotides (ASOs)

ASOs represent the first nucleic acid medications to be researched, and also approved for clinical applications [39]. ASOs have been studied since the 1980s in different research areas; however, in the late 1970s, Zamecnik and Stephenson described the ability of short synthetic oligonucleotides to inhibit the replication of Rous sarcoma virus by binding to its DNA, laying the groundwork for antisense technology [40]. Two decades later, Ionis Pharmaceuticals saw its first compound, fomivirsen (Vitravene), approved by the U.S. FDA. Since then, hundreds of ASO-based agents have been evaluated in clinical trials, marking significant progress in the field of RNA-targeted therapeutics [41]. Depending on their chemical modifications, ASOs are subdivided into three generations.

***Structure:*** ASOs are exogenous, single-stranded, synthetic oligodeoxyribonucleotides, typically short molecules comprising 15 to 30 nucleotides in length, capable of binding precisely with a complementary target mRNA sequence in a sequence-specific manner through the Watson–Crick base-pair interactions, and act via RNA cleavage or blockage [42]. The term “anti-sense” is used because its strands halt translation, while the genetic material being translated is referred to as the “sense” strand [43].

**Figure 2:**
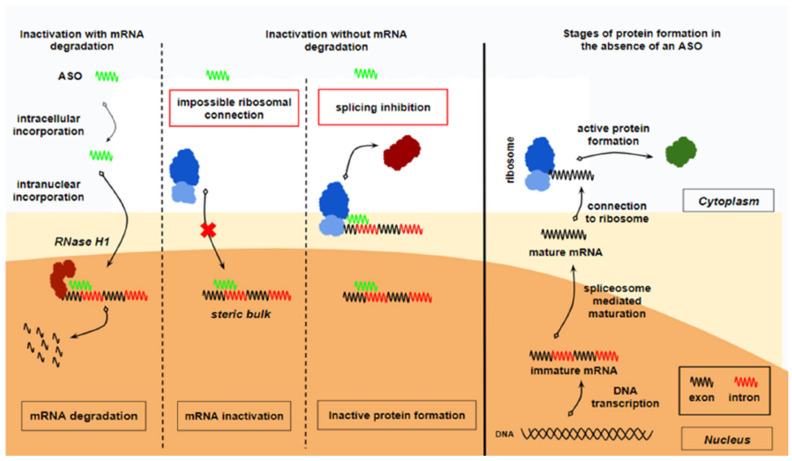
Mechanism of action of ASOs. Antisense oligonucleotides (ASOs) bind intranuclearly to the immature mRNA molecule and prevent the formation of an active protein. This occurs either via mRNA degradation or via the inactivation of the mRNA without degradation. Degradation of the mRNA is catalyzed by the enzyme RNase H1. Inactivation without either forming a steric bulk that prevents ribosome attachment to the mature mRNA or by splicing inhibition, which prevents the mRNA maturation process, thereby leading to the production of an inactive protein. Normally, in the absence of antisense oligonucleotides (ASOs), the enzymatic transcription of a DNA gene produces an immature mRNA molecule. This immature mRNA undergoes maturation through the action of the spliceosome, which removes introns, resulting in a mature mRNA molecule. The mature mRNA is then transported to the cytoplasm, where it binds to a ribosome and is translated into a protein. This process ultimately leads to the formation of an active protein. From “Novel RNA-Based Therapies in the Management of Dyslipidemias” by Kosmas CE et al., 2025, Int. J. Mol. Sci. 26, 1026, Figure 1 [46]. CC BY.

***Mechanism of action:*** ASOs inhibit the production of active proteins through the modification of their precursor mRNA molecule. This inhibition is mediated by mechanisms (Figure 2) that include binding complementarily to mRNA and subsequently: (a) inducing RNase-H-mediated degradation, (b) modulating mRNA splicing, (c) blocking translation through steric interference, and (d) stimulating an immune response mediated by multiple cytosine–phosphodiester–guanine sequences [44]. ASOs act in the nucleus, cytoplasm, or both, according to their specific mechanism of action [45].

ASOs require modifications in order to facilitate their uptake by target cells and prevent their degradation by endonucleases [46]. Chemical modifications, e.g., in backbone (phosphorothioate linkages and sugar and base modifications at the 2′ position) may alter the pharmacokinetic and pharmacodynamic properties, enhancing their functionality. Regarding cellular uptake, N-acetylgalactosamine (GalNAc) is an important conjugate that acts as a ligand for the asialoglycoprotein receptor (ASGPR) on hepatocytes, where cholesterol synthesis takes place. Binding to ASGPR allows for rapid internalization of the NAT-GalNAc complex in the afforementioned cells [44,45].

ANGPTL3-LRx, otherwise known as Vupanorsen, initially constituted an alternative, promising pharmacological agent to treat dyslipidemias. It is a second-generation ligand-conjugated ASO targeting the ANGPTL3 gene mRNA coding sequence in hepatocytes, ultimately inactivating its translation to ANGPTL3 protein.

Several preclinical and clinical studies have evaluated the role of ANGPTL3-LRx in dyslipidemia. A summary of the results of the main clinical trials discussed in this review is shown in Table 3.

In 2017, Graham et al. [47] evaluated ANGPTL3-LRx for its effects on plasma lipid levels, triglyceride clearance, liver triglyceride content, insulin sensitivity, and atherosclerosis in mice. They demonstrated that ANGPTL3-LRx use in mice effectively decreases levels of liver ANGPTL3 mRNA, ANGPTL3 protein, TG, and LDL-cholesterol in a dose-dependent manner, along with reductions in hepatic TG content and atherosclerosis progression. Subsequently, they properly designed a randomized, double-blind, placebo-controlled, Phase 1 clinical trial, funded by Ionis Pharmaceuticals (NCT02709850) enrolling 44 adult participants with an age ranging from 18 to 65, randomly assigned in a 3:1 ratio to receive placebo or ANGPTL3-LRx with up to 80 mg in a single dose or up to 60 mg weekly. This trial assessed the safety, possible adverse events, pharmacokinetics, and pharmacodynamics of single and multiple ascending doses of ANGPTL3-LRx. Findings were consistent with data derived from the preclinical mice trial; at day 43, ANGPTL3-LRx reduced the levels of ANGPTL3 protein, TG, LDL-cholesterol, ApoB, and non-HDL-cholesterol by up to 84.5%, 63.1%, 32.9%, 25.7%, and 36.6%, respectively, as compared with placebo. Except for headache and dizziness—reported by three participants who received the antisense oligonucleotide and three who received placebo—no serious adverse events were noted [47].

A multicenter, randomized, double-blind, placebo-controlled, dose-ranging Phase 2 study was conducted in 105 patients with hypertriglyceridemia, type 2 diabetes mellitus (T2DM), and nonalcoholic fatty liver disease (NAFLD) (NCT03371355). NCT03371355 to evaluate the safety of AKCEA-ANGPTL3-LRx and to assess the efficacy of different doses and dosing regimens of AKCEA-ANGPTL3-LRx on glucose and lipid metabolism, and liver fat. The primary endpoint of a significant reduction in triglyceride levels was achieved. Subjects were randomized in a 3:1 ratio to receive SC Vupanorsen or placebo for 6 months at doses of 40 or 80 mg every 4 weeks or 20 mg every week [48]. The primary efficacy endpoint was the percent change in fasting triglycerides levels from baseline at 6 months. The reduction in TG from baseline in patients receiving Vupanorsen reached a maximum of 53%, with 35–58% of the patients (depending on the administered dose regimen) achieving normal levels of plasma TGs. ANGPTL3 protein was markedly reduced by up to 62%. In addition, Vupanorsen, as compared with placebo, reduced apoC-III, remnant cholesterol, total cholesterol, non-HDL-cholesterol, HDL-cholesterol, LDL-cholesterol, and ApoB by 58%, 38%, 19%, 18%, 24%, 12%, and 9%, respectively. Once again, no serious adverse events were reported in this study besides injection-site pruritus and injection-site erythema [48].

To evaluate the appropriate dose(s) of Vupanorsen in cardiovascular risk reduction and in severe hypertriglyceridemia, a multicenter, Phase 2b, double-blind, placebo-controlled, parallel group study (TRANSLATE-TIMI 70) was conducted to provide data on efficacy, safety, tolerability, and pharmacokinetics of Vupanorsen administered subcutaneously at various doses and regimens in 286 statin-treated participants with dyslipidemia (elevated non-HDL-cholesterol and TG). Its primary endpoint was the percent change from baseline in non-HDL-cholesterol, with secondary endpoints being reduced in ANGPTL3, LDL-cholesterol, ApoB, and TG at weeks 16 and 24 [49]. Although the study met its primary endpoint, achieving a statistically significant reduction in non-HDL-cholesterol, TG, and ANGPTL3, the magnitude of the reduction in non-HDL-cholesterol and TG levels observed did not encourage the continuation of the study. Moreover, a dose-dependent elevation in hepatic fat, as well as in liver enzymes alanine aminotransferase (ALT) and aspartic aminotransferase (AST), was observed. According to Pfizer’s official press release, these events prompted Pfizer Inc. and Ionis Pharmaceuticals to discontinue the clinical development program of Vupanorsen, as announced on 31 January 2022 [50].

The development of ASOs at its inception faced many challenges, including problems with stability, delivery, and specificity, as the early ASOs were highly susceptible to degradation by nucleases present in biological fluids, thereby severely compromising their therapeutic efficacy.

#### 3.2.2. Small (Short) Interfering RNAs (siRNAs)

RNA interference is a molecular phenomenon with micro RNAs and some long noncoding RNAs exerting their function with exquisite specificity and efficiency by complementary base-pairing to their RNA targets [51]. The description of this molecular phenomenon allowed Andrew Z. Fire and Craig C. Mello to be awarded the Nobel Prize in Physiology or Medicine in 2006.

***Structure:*** The discovery of Small (Short) Interfering RNAs (siRNAs) dates back to 1998 when researchers were studying the mechanism of gene silencing in plants and found that small RNA molecules were responsible for this process, which they later named siRNAs. Since then, siRNAs have been identified in many organisms, including humans. siRNAs are RNA molecules that have a key role in gene regulation, as they can bind to complementary mRNA molecules blocking their translation into proteins. From the structure point of view, siRNAs are short, double-stranded RNA sequences composed of about twenty base pairs and consist of a passenger (sense) strand and a guide (antisense) strand, which are complementary to each other.

**Figure 3:**
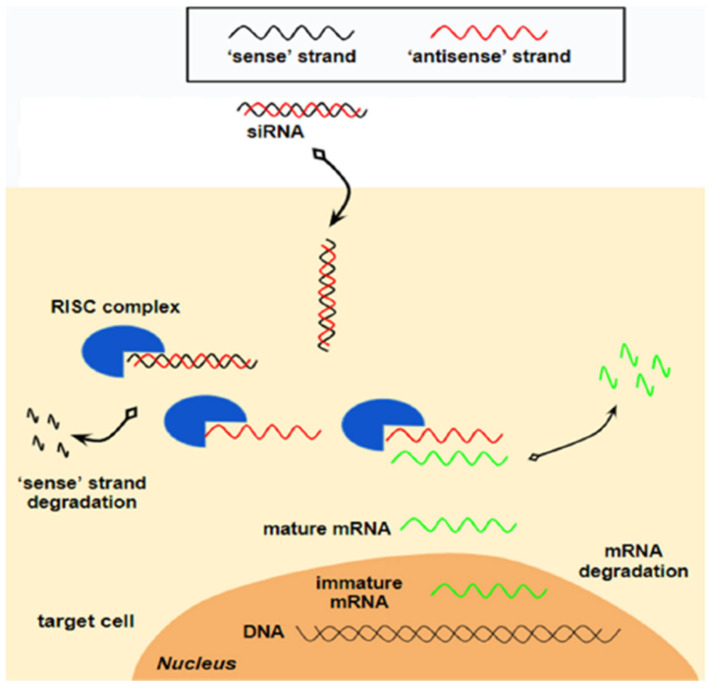
Mechanism of action of siRNAs. siRNAs consist of a double-stranded RNA molecule, containing a sense strand and an antisense strand. The antisense strand acts as a guide, facilitating the association of siRNA with a protein complex, called “the RISC” (RNA-induced silencing complex). Upon association with the RISC complex, the sense strand is degraded, while the antisense strand remains bound to the target mature mRNA. From “Novel RNA-Based Therapies in the Management of Dyslipidemias” by Kosmas CE et al., 2025, Int. J. Mol. Sci. 26, 1026, Figure 2 [46] (https://doi.org/10.3390/ijms26031026). CC BY.

***Mechanism of action:*** The double-stranded siRNA interacts with the RNA induced silencing complex (RISC), prompting the passenger strand to unwind and degrade, while the guide strand binds to the target mRNA, directing its RISC-mediated cleavage (Figure 3). Thus, the synthesis of the target protein is hindered [52].

More specifically, once siRNAs are internalized in the hepatocytes through the mediation of GalNac, one of the chains, acting as a guide in the intracellular path, directs the oligonucleotide construction towards the cell cytoplasm. Then, the guide chain separates and is degraded, while the antisense strand is incorporated into the RISC, where the translation of RNA into protein takes place. This antisense strand specifically locates the target RNA and conjugates with it, blocking the translation into protein, creating a target for the action of RNases that will degrade the target RNA [53].

This therapeutic mechanism has been used to develop therapies targeting PCSK9 (inclisiran), Lp(a), ANGPTL-3 and apo CIII in the cardiovascular field. In all cases, the siRNA molecules are conjugated with GalNac to ensure the specificity of their intrahepatocyte action.

Especially for ANGPTL3 gene that is expressed mainly in the liver, RNA interference-based therapeutic approaches inhibit hepatic translation of ANGPTL3 messenger RNA (mRNA), thus having the potential to modulate levels of atherogenic lipid parameters. Solbinsiran (LY3561774) and Zodasiran (ARO-ANG3) are two RNA interference therapeutics targeting hepatic ANGPTL3 mRNA.

Solbinsiran or LY3561774 is a N-acetylgalactosamine (GalNAc)-conjugated siRNA, which targets hepatic ANGPTL3 protein expression and is undergoing investigation in research settings [54]. In a Phase 1, multicenter, randomized, double-blind, placebo-controlled study (NCT04644809), Ray KK et al. evaluated the impact of solbinsiran to inhibit hepatic translation of ANGPTL3 mRNA, on ANGPTL3 and lipid levels in preclinical models and humans [55]. In mice expressing human ANGPTL3, a single dose of solbinsiran reduced hepatocyte ANGPTL3 mRNA by 65% compared to vehicle-treated mice. In cynomolgus monkeys, solbinsiran decreased hepatic ANGPTL3 mRNA by up to 73% and serum ANGPTL3 protein by up to 69%. In humans, a single dose of solbinsiran resulted in dose-dependent mean percentage reductions from baseline in ANGPTL3 up to 86%, TG up to 73%, LDL-cholesterol up to 30%, non-HDL-cholesterol up to 41%, and apoB up to 30%, with sustained effects at higher doses (*p* < 0.0001 for all). The repeat-dose study demonstrated reductions in ANGPTL3 of 89%, TG up to 70%, LDL-cholesterol up to 42%, non-HDL-cholesterol up to 46%, and apoB up to 36% (*p* < 0.0001 for all). Nuclear magnetic resonance lipoprotein analysis demonstrated that the solbinsiran administration resulted in reductions in the total number of TG-rich lipoprotein and LDL particles. Adverse events were mostly mild in severity, with similar incidence in solbinsiran- and placebo-treated participants. The authors concluded that solbinsiran inhibits hepatic ANGPTL3 translation and results in significant reductions in all atherogenic lipoproteins up to day 90 in patients mixed dyslipidemia [55].

In a Phase 2 study, which enrolled adult patients with mixed dyslipidaemia (NCT05256654), Ray KK et al. further assessed the durability and efficacy of solbinsiran in reducing concentrations of atherogenic lipoproteins, as well as tolerability, including hepatic fat [56]. Of 585 patients screened, 205 patients were enrolled and were randomly assigned to receive solbinsiran 100 mg (n = 30), solbinsiran 400 mg (n = 58), solbinsiran 800 mg (n = 59), or placebo (n = 58). At baseline, median concentrations were: apoB 111 mg/dL, TG: 2.64 mmol/L, and LDL-cholesterol 3.16 mmol/L. The placebo-adjusted percent change in apoB concentration from baseline on day 180 was −2.8% (*p* = 0.69) for solbinsiran 100 mg, −14.3% (*p* < 0.01) for solbinsiran 400 mg, and −8.3% (*p* = 0.14) for solbinsiran 800 mg. Solbinsiran administration was well tolerated, with a low incidence of adverse events. The number of patients with treatment-emergent adverse events was 18 of 30 patients in the solbinsiran 100 mg group, 30 of 58 patients in the solbinsiran 400 mg group, 26 of 59 patients in the solbinsiran 800 mg group, and 37 of 57 patients in the placebo group. The authors concluded that solbinsiran, at a dose of 400 mg, provided durable reductions in serum ANGPTL3 concentrations in adults with mixed dyslipidaemia, resulting in significant and sustained reductions in apoB, triglyceride, non-HDL cholesterol, and LDL-cholesterol concentrations, and it was well tolerated, with a reassuring hepatic safety profile [56].

Zodasiran (also known as ARO-ANG3), a siRNA drug, designed to silence ANGPTL3 mRNA in the liver reducing ANGPTL3 protein levels, is currently under evaluation. The first-in-human, Phase 1, randomized, placebo-controlled, open-label trial (NCT03747224) conducted by Watts GF et al. [57], in order to evaluate safety and pharmacokinetics and pharmacodynamics of single and multiple doses of ARO-ANG3 in four cohorts of 52 healthy participants and one cohort of 9 participants with hepatic steatosis, was part of a basket trial. ARO-ANG3 was well tolerated, with similar frequencies of treatment-emergent adverse events in active and placebo groups. The authors found that the systemic absorption of ARO-ANG3 in healthy participants was rapid and sustained, with a mean T_max_ of 6.0–10.5 h and clearance from plasma within 24–48 h after dosing with a mean t_½_ of 3.9–6.6 h. In healthy participants, ARO-ANG3 treatment reduced ANGPTL3 (mean −45% to −78%) 85 days after dose. They observed reductions in levels of triglycerides (median −34% to −54%) and non-HDL-cholesterol (mean −18% to −29%) (exploratory endpoints) with the three highest doses and concluded that these early-phase data support ANGPTL3 as a potential therapeutic target for ASCVD management [57].

Rosenson RS et al. conducted the ARCHES-2 study (NCT04832971), a double-blind, placebo-controlled, dose-ranging Phase 2b trial to evaluate the safety and efficacy of zodasiran in adults with mixed hyperlipidemia [58]. A total of 204 patients underwent randomization and received subcutaneous injections of zodasiran (50, 100, or 200 mg) or placebo on day 1 and week 12 and were followed through week 36. The authors reported that at week 24, the administration of zodasiran resulted in substantial mean dose-dependent decreases from baseline in ANGPTL3 levels (difference vs. placebo, −54% with 50 mg, −70% with 100 mg, and −74% with 200 mg), and significant dose-dependent decreases in triglyceride levels (difference vs. placebo, −51%, −57%, and −63%, respectively) (*p* < 0.001 for all comparisons). Other differences from baseline as compared with placebo included the following: for non-HDL cholesterol level, −29% with 50 mg, −29% with 100 mg, and −36% with 200 mg; for apolipoprotein B level, −19%, −15%, and −22%, respectively; and for LDL-cholesterol level, −16%, −14%, and −20%, respectively. Of note, a transient elevation in glycated hemoglobin levels in patients with preexisting diabetes who received the highest dose of zodasiran was also observed [58]. The authors concluded that in patients with mixed hyperlipidemia, zodasiran was associated with significant decreases in triglyceride levels at 24 weeks.

Another Phase 2, open label clinical trial (NCT05217667) runs from April 2022 to November 2025 and involves participants with HoFH who will receive two open-label doses of ARO-ANG3 and be evaluated for safety and efficacy parameters through 36 weeks. According to the trial description, participants who will complete the first 36 week treatment period may opt to continue in an additional 24 month extension period during which they will receive up to eight doses open-label doses of ARO-ANG3 [59].

A summary of the key clinical trials of the novel RNA-based therapies discussed in this review is shown in Table 4.

## 4. Promising Gene Editing for Dyslipidemias

Gene editing targets the genetically regulated pathways, holding great promise for the treatment of cardiometabolic diseases. Nucleases or other engineered proteins are used to induce a permanent alteration of gene expression, thereby potentially also altering the susceptibility to disease. Gene editing of genes regulating lipid metabolism including PCSK9, ANGPTL3, ANGPTL4, APOC3 (apolipoprotein C-III), and LPA, holds promise as an exciting new therapeutic approach [60].

Gene editing technologies such as Clustered Regularly Interspaced Short Palindromic Repeats (CRISPR)/Cas systems, base editing, prime editing, and epigenome editing, have revolutionized the ability to precisely manipulate genomic sequences for therapeutic purposes [61]. Among them, base editing and CRISPR nuclease editing are two revolutionary techniques in gene editing. Particularly, CRISPR-associated protein 9 (Cas9) gene editing has emerged as one of the most promising biotechnological tools in our time because of its high efficiency and relative simplicity [62,63]. CRISPR-Cas9 technology is currently recognized as providing the most efficient programmable nucleases for precisely inducing double-strand breaks at targeted genomic locations in nuclear DNA. The CRISPR-Cas9 complex consists of the Cas9 enzyme, which acts as a nuclease capable of cleaving both DNA strands and a single guide RNA (sgRNA) that directs Cas9 to the targeted genomic site. Of note, the CRISPR-Cas9 system can inactivate genes implicated in cardiovascular and metabolic diseases, with base editing providing a potential advantage in reducing off-target effects and enhancing safety profiles [62].

Recently, CRISPR-based therapies have been used to permanently inactivate cholesterol-related genes in the liver. In 2023, CRISPR Therapeutics began two Phase 1 trials: CTX320 targeted the LPA gene and CTX310^TM^ designed to knock out hepatic expression of ANGPTL3, the gene that encodes a protein that regulates levels of low-density lipoproteins (LDLs) and triglycerides (TGs). CTX310 is a CRISPR-Cas9 gene-editing therapy comprising Cas9 mRNA and a guide RNA targeting ANGPTL3, encapsulated into lipid nanoparticles (LNPs) for targeted delivery to the liver. A Cas9 nuclease is used to disrupt the target gene by imparting a double-stranded break in the DNA rather than editing a specific nucleotide base using a base editor.

Preclinical data showed that a single dose of CTX310 in non-human primates resulted in 70% mean editing of ANGPTL3 in the liver, an over 85% reduction in plasma ANGPTL3 protein, and a 60% reduction in TG, with effects lasting beyond a year and only transient liver enzyme elevation [64]. The ongoing Phase I first-in-human clinical trial is targeting ANGPTL3 in four patient groups: homozygous familial hypercholesterolemia (HoFH), severe hypertriglyceridemia (sHTG), heterozygous familial hypercholesterolemia (HeFH), or mixed dyslipidemias (MDL). New Phase 1 clinical data for CTX310^TM^ continues to demonstrate dose-dependent reductions in TG and LDL, with peak reduction of up to 82% in TG and up to 86% in LDL, with a well-tolerated safety profile. The complete Phase 1 data presentation for CTX310 anticipated at a medical meeting in the second half of 2025.

A few days ago Laffin LJ et al. published in N. Engl. J. Med. [65] that editing of ANGPTL3 was associated with few adverse events and resulted in reductions from baseline in ANGPTL3 levels. The authors conducted an ascending-dose Phase 1 trial to assess the safety and efficacy of CTX310, a lipid-nanoparticle-encapsulated CRISPR-Cas9 mRNA and guide RNA targeting hepatic *ANGPTL3* to induce a loss-of-function mutation. A total of 15 participants received CTX310 and had at least 60 days of follow-up. No dose-limiting toxic effects related to CTX310 occurred. Serious adverse events occurred in two participants (13%) (one participant had a spinal disk herniation, and the other died suddenly 179 days after treatment with the 0.1 mg-per-kilogram dose). Moreover, infusion-related reactions were reported in three participants (20%), and one participant (7%) who had elevated levels of aminotransferases at baseline had a transient elevation in aminotransferases between three times and five times as high as those at baseline, peaking on day 4 and returning to baseline by day 14. The mean % change in ANGPTL3 level was 9.6% with the dose of 0.1 mg per kilogram, 9.4% with 0.3 mg per kilogram, −32.7% with 0.6 mg per kilogram, −79.7% with 0.7 mg per kilogram, and −73.2% with 0.8 mg per kilogram [65].

Rapid advances in RNA therapies and genome editing offer great promise for research and medicine but also raise biosafety and ethical concerns. However, with the widespread application of genome-editing technologies, biosafety issues have gradually attracted the attention of the scientific community and the public. Potential risks such as off-target effects, genomic instability, and ethical and legal issues need to be taken seriously. Genome-editing may lead to unexpected genetic mutations, which in turn can cause unknown health problems. The ethical and legal challenges associated with human genome editing are notably intricate and multifaceted. Robust international and national regulatory frameworks are essential to safeguard the responsible and secure implementation of genome-editing technologies. Future research needs to further improve the accuracy of genome-editing tools, reduce off-target effects and unexpected mutations, and develop a robust regulatory framework. Moreover, enhancing public education and outreach to increase awareness and understanding of genome-editing technologies can facilitate the development of social consensus and minimize ethical controversies in its application. Through these measures, genome-editing technologies are expected to achieve safe, effective, and responsible applications in the future, bringing more benefits to human health and scientific development [66].

## 5. Conclusions—Future Directions

The therapeutic landscape of lipid disturbances that is focused on the regulation and modulation of pathways underlying lipoprotein metabolism disorders is undergoing rapid and significant expansion. The identification of new therapeutic targets based on clinical and basic research and supported by genetic data has provided the opportunity of acting on residual cardiovascular risk beyond the reduction of LDL-cholesterol levels.

Inhibition of ANGPTL3 is a promising therapeutic target for the management of dyslipidemia, a major etiology behind cardiovascular disorders. In clinical practice, monoclonal antibody drug, e.g., evinacumab, which targets, binds to, and pharmacologically inhibits ANGPTL3, demonstrated substantial therapeutic effects. Of note, new lipid-lowering therapies that target ANGPTL3 are progressing. The cutting-edge RNA-based therapies, including ASOs and siRNAs, primarily target ANGPTL3 and represent a significant advancement in the treatment of lipid disorders. The results derived from clinical trials evaluating these RNA therapies have been positive, fueling optimism for their future integration into clinical practice. As these therapies demonstrate promising efficacy, they are expected to significantly improve the management of dyslipidemias and reduce cardiovascular risk, a critical concern for patients with lipid abnormalities. Genome editing of ANGPTL3 gene with CRISPR-Cas9 technology offers an innovative and promising approach to treating CVDs by precisely targeting the genetic mutations underlying lipid disorders while also being employed to modulate the expression of genes that contribute to CVD progression.

However, these approaches face critical challenges (off-target effects, ethical concerns, possible misuse, unintended risks, and cost) that should be addressed, as they are crucial for translating laboratory success into clinical practice. Evaluation of the long-term safety, effectiveness, and interaction of these therapies with other chronic conditions and medical treatments, as in the case of diabetes mellitus and hypertension, is necessary. Clear rules and guidelines are needed to ensure they are used safely. Another key focus should be investigating how these therapies work alongside traditional therapeutic agents like statins, fibrates, or PCSK9 inhibitors to obtain the best results for dyslipidemia patients. Data is very encouraging; however, further studies are needed to introduce those new molecules into everyday clinical practice.

## Figures and Tables

**Table 1:** Novel ANGPTL3 targeting therapies.

Therapy	Mechanism	Key LDL-C/TG Reduction (Trials)	Safety Concerns	Status (2025)
**Evinacumab**	Binds/inhibits ANGPTL3 protein	LDL-C: 43–59% TG: 47% (ELIPSE HoFH, n = 64)	Infusion reactions (3–16%)	FDA/EMA-approved for HoFH ≥ 12 y
**Zodasiran** (ARO-ANG3)	Blocks gene mRNA transcripts	TG: 63% LDL-C by 20%	Transient glycemic changes	Phase 2
**Solbinsiran (LY3561774)**	Blocks gene mRNA transcripts	TG: 73%, LDL-C: 30%	Well-tolerated	Phase 2
**CTX310 (CRISPR/Cas9)**	DSB-induced knockout	TG: 82%; LDL-C: 86% (Phase 1, n = 15)	Transient ALT ↑ (1/15)	Phase 1 ongoing
**SHR1918**	Binds/inhibits ANGPTL3 protein	LDL-C: 21.7% to 29.9% (Phase 2, NCT06109831)	Well-tolerated	Phase 2

**Table 3:** Summary of the results of the clinical studies pertaining to antisense oligonucleotides (ASOs), Vupanorsen.

	Clinical Trial	Study Design	Results
Graham et al. *The New England Journal of Medicine* **2017** [47]	Phase 1 NCT02709850	Double-blind, placebo-controlled, Phase 1 clinical trial 44 healthy volunteers (TGs 90–150 mg/dL) 20, 40, o 80 mg subcutaneous	TG: reduction 33.2–63.1% LDL-cholesterol: reduction 1.3–32.9% VLDL-cholesterol: reduction 27.9–60.0% Non-HDL-cholesterol: reduction 10–36% ApoB: reduction 3.4–25.7% ApoCIII: reduction 18.9–58.8%
Gaudet D et al. *European Heart Journal* **2020** [48]	Phase 2 NCT03371355	Randomized, double-blind, placebo-controlled, multicenter, dose-ranging study 105 patients having TGs > 150 mg/dL, T2DM, hepatic steatosis and a body mass index (BMI) between 27 and 40 kg/m^2^ 6 months treatment 40 or 80 mg every 4 weeks or 20 mg weekly.	TG reduction 36% with 40 mg every 4 weeks reduction 53% with 80 mg every 4 weeks reduction 47% with 20 mg weekly Total Cholesterol reduction 19% with 80 Q4W “remnant” cholesterol reduction 38% with 80 mg every 4 weeks non-HDL-C reduction 18% with 80 mg every 4 weeks

**Table 4:** Summary of the results of the clinical studies pertaining to small (short) interfering RNAs (siRNAs).

	Clinical Trial	Study Design	Results
**Solbinsiran (LY3561774)**			
Ray KK et al. *J. Am. Coll. Cardiol.* **2025** [55]	Phase 1-NCT04644809	Patients with mixed dyslipidemia. Single subcutaneous doses of solbinsiran (24–960 mg) or matching placebo. Repeat dose study, subcutaneous solbinsiran (208 or 480 mg) or matching placebo on days 1 and 29 was evaluated.	Dose-dependent mean % reductions in ANGPTL3 up to 86%, TG up to 73%, LDL-cholesterol up to 30%, non-HDL-cholesterol up to 41%, and apoB up to 30%. Repeat-dose study: reductions in ANGPTL3 of 89%, TG up to 70%, LDL-cholesterol up to 42%, non-HDL-cholesterol up to 46%, and apoB up to 36%.
Ray KK et al. *Lancet* **2025** [56]	Phase 2-NCT05256654	Phase 2b, multicenter, double-blind, placebo-controlled, parallel-group (175 mixed dyslipidemia patients) Solbinsiran (three dosage regimens 100 mg, 400 mg, 800 mg) vs. placebo.	Solbinsiran, at a dose of 400 mg, provided durable reductions in serum ANGPTL3 concentrations in adults with mixed dyslipidaemia. Significant and sustained reductions in apoB, TG, non-HDL cholesterol, and LDL-cholesterol. Solbinsiran administration was well tolerated, with a low incidence of adverse events.
**Zodasiran (ARO-ANG3)**			
Watts GF et al. *Nat. Med.* **2023** [57]	Phase 1-NCT03747224	Phase 1, randomized, placebo-controlled, open-label trial. One cohort of 9 participants with hepatic steatosis vs. 52 healthy participants	Reduction in ANGPTL3 (mean −45% to −78%) 85 days after dose. Reductions in TG (median −34% to −54%) and non-HDL-cholesterol (mean −18% to −29%). Well tolerated.
Rosenson RS et al. *N. Engl. J. Med.* **2024** [58]	ARCHES-2 Phase 2b-NCT04832971	Phase 2, dose-ranging, double-blind, randomized, placebo-controlled (204 patients, mixed hyperlipidemia) Zodasiran (50, 100, or 200 mg SC on day 1 and week 12) vs. placebo	At week 24: TG reduced by 63%, ANGPTL3 by 73.7%, non-HDL-C by 36.4%, LDL-C by 20%, and Lp(a) by 20%. No hepatic fat increase; transient HbA1C elevation. Well tolerated.
*Study of ARO-ANG3 in Participants with Homozygous Familial Hypercholesterolemia (HOFH) (Gateway)* [59]	Phase 2-NCT05217667	Phase 2, open label clinical trial (18 patients; homozygous familial hypercholesterolemia (HoFH) LDL-C > 100 mg/dL) Zodasiran (SC administration for 36 weeks, followed by up to eight open-label doses in a 24 month extension).	Participants who will complete the first 36 week treatment period may opt to continue in an additional 24 month extension period during which they will receive up to 8 open-label doses of ARO-ANG3.

## Data Availability

No new data were created or analyzed in this study. Data sharing is not applicable to this article.
